# 1-(5-Nitro-2-oxoindolin-3-yl­idene)thio­semicarbazide

**DOI:** 10.1107/S1600536811040293

**Published:** 2011-10-05

**Authors:** Katlen C. T. Bandeira, Leandro Bresolin, Johannes Beck, Jörg Daniels, Adriano Bof de Oliveira

**Affiliations:** a Escola de Química e Alimentos, Universidade Federal do Rio Grande, Av. Itália km 08, Campus Carreiros, 96201-900 Rio Grande, RS, Brazil; b Institut für Anorganische Chemie, Universität Bonn, Gerhard-Domagk-Strasse 1, D-53121 Bonn, Germany; c Departamento de Química, Universidade Federal de Sergipe, Av. Marechal Rondon s/n, Campus, 49100-000 São Cristóvão, SE, Brazil

## Abstract

In the title molecule, C_9_H_7_N_5_O_3_S, there is an intramolecular N—H⋯O. The molecule is essentially planar, with the maximum deviation from the mean plane of the 18 non-H atoms being 0.135 (2) Å for the amine N atom. In the crystal, the molecules are connected *via* intermolecular N—H⋯O and N—H⋯S hydrogen bonds, forming two-dimensional networks lying parallel to (10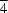). They are separated by an interplanar distance of 3.3214 (9) Å, leading to π–π interactions which stabilize the crystal structure.

## Related literature

For the pharmacological properties of isatine-thio­semi­carb­azone derivatives, including the title compound, against cruzain, falcipain-2 and rhodesain, see: Chiyanzu *et al.* (2003 ▶). For the synthesis of 5-nitro­isatine-3-thio­semi­carbazone, see: Campaigne & Archer (1952 ▶). For an example of a similar structure, 5-bromo­isatin-thio­semicarbazone, see: Pederzolli *et al.* (2011 ▶).
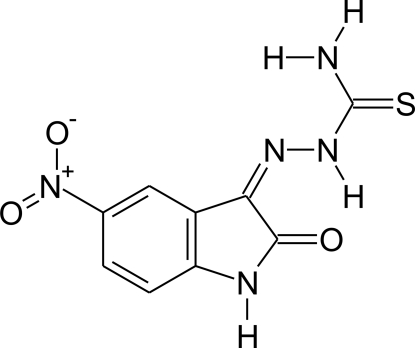


## Experimental

### 

#### Crystal data

C_9_H_7_N_5_O_3_S
*M* *_r_* = 265.26
Monoclinic, 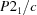

*a* = 5.2112 (2) Å
*b* = 15.5354 (5) Å
*c* = 13.8711 (5) Å
β = 105.855 (2)°
*V* = 1080.25 (7) Å^3^
*Z* = 4
Mo *K*α radiation
μ = 0.31 mm^−1^
*T* = 293 K
0.08 × 0.07 × 0.03 mm

#### Data collection

Nonius KappaCCD diffractometer
Absorption correction: analytical (Alcock, 1970 ▶) *T*_min_ = 0.966, *T*_max_ = 0.983
15688 measured reflections
2469 independent reflections
1646 reflections with *I* > 2σ(*I*)
*R*_int_ = 0.055

#### Refinement

*R*[*F* ^2^ > 2σ(*F* ^2^)] = 0.041
*wR*(*F* ^2^) = 0.108
*S* = 1.02
2469 reflections
191 parameters
All H-atom parameters refined
Δρ_max_ = 0.18 e Å^−3^
Δρ_min_ = −0.27 e Å^−3^

### 

Data collection: *COLLECT* (Nonius, 1998 ▶); cell refinement: *DENZO* and *SCALEPACK* (Otwinowski & Minor, 1997 ▶); data reduction: *DENZO* and *SCALEPACK*; program(s) used to solve structure: *SHELXS97* (Sheldrick, 2008 ▶); program(s) used to refine structure: *SHELXL97* (Sheldrick, 2008 ▶); molecular graphics: *ORTEP-3 for Windows* (Farrugia, 1997 ▶); software used to prepare material for publication: *WinGX* (Farrugia, 1999 ▶).

## Supplementary Material

Crystal structure: contains datablock(s) I, global. DOI: 10.1107/S1600536811040293/vm2117sup1.cif

Structure factors: contains datablock(s) I. DOI: 10.1107/S1600536811040293/vm2117Isup2.hkl

Supplementary material file. DOI: 10.1107/S1600536811040293/vm2117Isup3.cml

Additional supplementary materials:  crystallographic information; 3D view; checkCIF report

## Figures and Tables

**Table 1:** Hydrogen-bond geometry (Å, °)

*D*—H⋯*A*	*D*—H	H⋯*A*	*D*⋯*A*	*D*—H⋯*A*
N4—H5⋯O1	0.93 (2)	2.08 (2)	2.791 (2)	132.6 (19)
N5—H6⋯O1^i^	0.83 (2)	2.13 (3)	2.957 (2)	173 (2)
N5—H7⋯O2^ii^	0.90 (3)	2.36 (3)	3.215 (3)	160 (2)
N1—H4⋯S^iii^	0.88 (3)	2.45 (3)	3.3123 (18)	170 (2)

Symmetry codes: (i) 
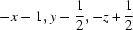
; (ii) 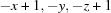; (iii) 
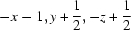
.

**Fig. 1.**
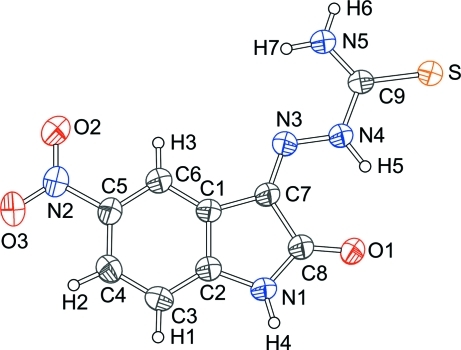
: The molecular structure of the title compound with labeling and displacement ellipsoids drawn at the 50% probability level.

**Fig. 2.**
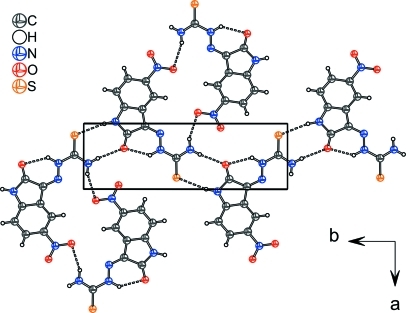
: Crystal structure of the title compound viewed in the direction of the crystallographic c-axis. Hydrogen bonding is indicated as dashed lines. The graphical representation is simplified for clarity.

## supplementary crystallographic information

### Comment

Thiosemicarbazone derivatives have a wide range of biological properties. For example, isatin-based synthetic thiosemicarbazones show pharmacological activity against cruzain, falcipain-2 and rhodesain (Chiyanzu *et al.*, 2003). As part of our study of thiosemicarbazone derivatives, we report herein the crystal structure of 5-nitroisatin-3-thiosemicarbazone. In the title compound (Fig. 1), the 5-nitroisain-3-thiosemicarbazone unit is planar and the maximal deviation from the least squares plane through all 18 non-hydrogen atoms is observed for N5 (0.135 (2) Å). The best plane through the thiosemicarbazide group (maximal deviation of 0.029 (2) Å for N4) makes an angle of 5.91 (8)° with the best plane through the isatine group (maximal deviation of 0.008 (2) Å for atoms C2, C4, C7). The nitro group is coplanar with the isatine ring (O2—N2—C5—C6 -0.7°). The bond angles suggest *sp*^2^ hybridization for the C and N atoms and explain the planarity of the title compound. The crystal packing is stabilized by intermolecular N—H···O and N—H···S (Table 1; N5-H6···O1^i^, N5-H7···O2^ii^, N1-H4···S^iii^) and intramolecular N—H···O1 bonds (Table 1; N4-H5···O1), building a two-dimensional H-bonded network (Fig. 2). The crystal packing is also stabilised by aromatic π–π-interactions between the isatine-thiosemicarbazone derivative molecules. The idealized plane through all 18 non-hydrogen atoms of adjacent molecules have an interplanar distance of 3.3214 (9) Å and are parallel. Symmetry codes: (i) -x-1, y-1/2, -z+1/2; (ii) -x+1, -y, -z+1; (iii) -x-1, y+1/2, -z+1/2.

### Experimental

Starting materials were commercially available and were used without further purification. The synthesis was adapted from a procedure reported previously (Campaigne & Archer, 1952). The hydrochloric acid catalyzed reaction of 5-nitroisatin (5,2 mmol) and thiosemicarbazide (5,2 mmol) in ethanol (60 ml) was refluxed for 6 h. After cooling and filtering, crystals suitable for X-ray diffraction were obtained.

### Refinement

All hydrogen atoms were localized in a difference density Fourier map. Their positions and isotropic displacement parameters were refined.

### Crystal data

**Table:**

C_9_H_7_N_5_O_3_S	*F*(000) = 544
*M**_r_* = 265.26	*D*_x_ = 1.631 Mg m^−^^3^
Monoclinic, *P*2_1_/*c*	Mo *K*α radiation, λ = 0.71073 Å
Hall symbol: -P 2ybc	Cell parameters from 26694 reflections
*a* = 5.2112 (2) Å	θ = 2.9–27.5°
*b* = 15.5354 (5) Å	µ = 0.31 mm^−^^1^
*c* = 13.8711 (5) Å	*T* = 293 K
β = 105.855 (2)°	Prism, colourless
*V* = 1080.25 (7) Å^3^	0.08 × 0.07 × 0.03 mm
*Z* = 4	

### Data collection

**Table:**

Nonius KappaCCD diffractometer	2469 independent reflections
Radiation source: fine-focus sealed tube	1646 reflections with *I* > 2σ(*I*)
graphite	*R*_int_ = 0.055
Detector resolution: 9 pixels mm^-1^	θ_max_ = 27.5°, θ_min_ = 3.0°
CCD rotation images, thick slices scans	*h* = −6→6
Absorption correction: analytical (Alcock, 1970)	*k* = −19→20
*T*_min_ = 0.966, *T*_max_ = 0.983	*l* = −18→17
15688 measured reflections	

### Refinement

**Table:**

Refinement on *F*^2^	Primary atom site location: structure-invariant direct methods
Least-squares matrix: full	Secondary atom site location: difference Fourier map
*R*[*F*^2^ > 2σ(*F*^2^)] = 0.041	Hydrogen site location: difference Fourier map
*wR*(*F*^2^) = 0.108	All H-atom parameters refined
*S* = 1.02	*w* = 1/[σ^2^(*F*_o_^2^) + (0.0502*P*)^2^ + 0.2777*P*] where *P* = (*F*_o_^2^ + 2*F*_c_^2^)/3
2469 reflections	(Δ/σ)_max_ < 0.001
191 parameters	Δρ_max_ = 0.18 e Å^−^^3^
0 restraints	Δρ_min_ = −0.27 e Å^−^^3^

### Special details

**Table:**

Geometry. All e.s.d.'s (except the e.s.d. in the dihedral angle between two l.s. planes) are estimated using the full covariance matrix. The cell e.s.d.'s are taken into account individually in the estimation of e.s.d.'s in distances, angles and torsion angles; correlations between e.s.d.'s in cell parameters are only used when they are defined by crystal symmetry. An approximate (isotropic) treatment of cell e.s.d.'s is used for estimating e.s.d.'s involving l.s. planes.
Refinement. Refinement of *F*^2^ against ALL reflections. The weighted *R*-factor *wR* and goodness of fit *S* are based on *F*^2^, conventional *R*-factors *R* are based on *F*, with *F* set to zero for negative *F*^2^. The threshold expression of *F*^2^ > σ(*F*^2^) is used only for calculating *R*-factors(gt) *etc*. and is not relevant to the choice of reflections for refinement. *R*-factors based on *F*^2^ are statistically about twice as large as those based on *F*, and *R*- factors based on ALL data will be even larger.

### Fractional atomic coordinates and isotropic or equivalent isotropic displacement parameters (Å^2^)

**Table:**

	*x*	*y*	*z*	*U*_iso_*/*U*_eq_	
S	−0.82185 (11)	0.04442 (3)	0.13953 (5)	0.0550 (2)	
O1	−0.3758 (3)	0.30256 (9)	0.25265 (11)	0.0497 (4)	
O2	0.8282 (4)	0.06044 (11)	0.57143 (16)	0.0833 (6)	
O3	1.0641 (3)	0.17173 (11)	0.62922 (13)	0.0689 (5)	
N1	0.0324 (3)	0.34347 (11)	0.35807 (13)	0.0456 (4)	
H4	−0.001 (5)	0.3986 (19)	0.3598 (18)	0.068 (8)*	
N2	0.8602 (4)	0.13826 (13)	0.57750 (14)	0.0534 (5)	
N3	−0.1521 (3)	0.12472 (10)	0.31461 (13)	0.0416 (4)	
N4	−0.3969 (3)	0.12302 (10)	0.24907 (13)	0.0438 (4)	
H5	−0.481 (5)	0.1742 (16)	0.2237 (17)	0.059 (7)*	
N5	−0.3935 (4)	−0.02231 (12)	0.26970 (16)	0.0564 (5)	
H6	−0.469 (5)	−0.0697 (16)	0.2592 (18)	0.059 (7)*	
H7	−0.234 (6)	−0.0184 (18)	0.315 (2)	0.072 (8)*	
C1	0.2206 (4)	0.21247 (12)	0.40888 (14)	0.0382 (4)	
C2	0.2575 (4)	0.30160 (12)	0.41775 (15)	0.0407 (5)	
C3	0.4895 (4)	0.33798 (14)	0.47706 (16)	0.0473 (5)	
H1	0.519 (5)	0.3964 (16)	0.4829 (16)	0.055 (6)*	
C4	0.6870 (4)	0.28252 (14)	0.52923 (16)	0.0470 (5)	
H2	0.847 (5)	0.3009 (15)	0.5747 (17)	0.058 (6)*	
C5	0.6461 (4)	0.19440 (13)	0.52072 (15)	0.0423 (5)	
C6	0.4156 (4)	0.15681 (13)	0.46118 (16)	0.0430 (5)	
H3	0.394 (4)	0.0988 (15)	0.4573 (16)	0.050 (6)*	
C7	−0.0416 (4)	0.19895 (12)	0.33974 (14)	0.0398 (5)	
C8	−0.1545 (4)	0.28607 (12)	0.30925 (15)	0.0416 (5)	
C9	−0.5237 (4)	0.04538 (12)	0.22445 (15)	0.0420 (5)	

### Atomic displacement parameters (Å^2^)

**Table:**

	*U*^11^	*U*^22^	*U*^33^	*U*^12^	*U*^13^	*U*^23^
S	0.0442 (3)	0.0362 (3)	0.0713 (4)	−0.0030 (2)	−0.0065 (3)	0.0049 (2)
O1	0.0429 (8)	0.0369 (7)	0.0590 (9)	0.0058 (6)	−0.0035 (7)	−0.0004 (6)
O2	0.0670 (12)	0.0471 (10)	0.1111 (15)	0.0072 (8)	−0.0173 (10)	0.0094 (10)
O3	0.0427 (9)	0.0704 (11)	0.0784 (12)	−0.0001 (8)	−0.0093 (8)	0.0074 (9)
N1	0.0454 (10)	0.0282 (8)	0.0561 (11)	0.0011 (7)	0.0019 (8)	−0.0007 (7)
N2	0.0430 (10)	0.0537 (12)	0.0571 (11)	0.0054 (8)	0.0030 (9)	0.0054 (9)
N3	0.0381 (9)	0.0343 (8)	0.0480 (9)	−0.0002 (7)	0.0045 (7)	−0.0019 (7)
N4	0.0398 (9)	0.0309 (8)	0.0532 (10)	0.0015 (7)	0.0002 (8)	−0.0010 (7)
N5	0.0464 (11)	0.0318 (9)	0.0769 (14)	−0.0018 (8)	−0.0072 (10)	0.0044 (9)
C1	0.0392 (10)	0.0314 (9)	0.0416 (10)	−0.0009 (8)	0.0070 (8)	−0.0015 (8)
C2	0.0405 (11)	0.0342 (10)	0.0451 (11)	0.0008 (8)	0.0080 (9)	−0.0011 (8)
C3	0.0484 (12)	0.0355 (11)	0.0541 (13)	−0.0056 (9)	0.0076 (10)	−0.0060 (9)
C4	0.0415 (11)	0.0466 (12)	0.0483 (12)	−0.0038 (9)	0.0047 (10)	−0.0050 (9)
C5	0.0378 (10)	0.0432 (11)	0.0427 (11)	0.0043 (8)	0.0055 (9)	0.0028 (8)
C6	0.0420 (11)	0.0354 (10)	0.0485 (12)	0.0011 (8)	0.0073 (9)	−0.0002 (9)
C7	0.0412 (11)	0.0304 (9)	0.0446 (11)	0.0024 (8)	0.0063 (9)	−0.0011 (8)
C8	0.0406 (11)	0.0344 (10)	0.0463 (11)	0.0014 (8)	0.0058 (9)	−0.0013 (8)
C9	0.0404 (10)	0.0317 (9)	0.0506 (12)	0.0006 (8)	0.0069 (9)	−0.0014 (8)

### Geometric parameters (Å, °)

**Table:**

S—C9	1.674 (2)	N5—H6	0.83 (2)
O1—C8	1.231 (2)	N5—H7	0.90 (3)
O2—N2	1.220 (2)	C1—C6	1.380 (3)
O3—N2	1.224 (2)	C1—C2	1.399 (3)
N1—C8	1.357 (3)	C1—C7	1.454 (3)
N1—C2	1.398 (2)	C2—C3	1.384 (3)
N1—H4	0.88 (3)	C3—C4	1.385 (3)
N2—C5	1.464 (3)	C3—H1	0.92 (2)
N3—C7	1.294 (2)	C4—C5	1.386 (3)
N3—N4	1.350 (2)	C4—H2	0.94 (2)
N4—C9	1.373 (2)	C5—C6	1.387 (3)
N4—H5	0.93 (2)	C6—H3	0.91 (2)
N5—C9	1.314 (3)	C7—C8	1.490 (3)
			
C8—N1—C2	111.20 (17)	C2—C3—H1	123.6 (15)
C8—N1—H4	122.3 (17)	C4—C3—H1	118.9 (15)
C2—N1—H4	125.5 (17)	C3—C4—C5	119.68 (19)
O2—N2—O3	122.80 (18)	C3—C4—H2	123.9 (14)
O2—N2—C5	118.90 (18)	C5—C4—H2	116.3 (14)
O3—N2—C5	118.30 (19)	C4—C5—C6	123.69 (19)
C7—N3—N4	117.92 (16)	C4—C5—N2	117.76 (18)
N3—N4—C9	119.14 (16)	C6—C5—N2	118.55 (18)
N3—N4—H5	119.9 (15)	C1—C6—C5	116.30 (19)
C9—N4—H5	120.9 (15)	C1—C6—H3	122.0 (14)
C9—N5—H6	117.8 (17)	C5—C6—H3	121.7 (14)
C9—N5—H7	122.6 (18)	N3—C7—C1	125.12 (17)
H6—N5—H7	119 (2)	N3—C7—C8	128.38 (17)
C6—C1—C2	120.68 (17)	C1—C7—C8	106.45 (15)
C6—C1—C7	132.90 (17)	O1—C8—N1	126.92 (18)
C2—C1—C7	106.43 (16)	O1—C8—C7	126.75 (17)
C3—C2—N1	128.15 (18)	N1—C8—C7	106.32 (16)
C3—C2—C1	122.23 (18)	N5—C9—N4	115.68 (18)
N1—C2—C1	109.61 (16)	N5—C9—S	126.01 (16)
C2—C3—C4	117.42 (19)	N4—C9—S	118.29 (14)
			
C7—N3—N4—C9	−177.19 (19)	C7—C1—C6—C5	179.5 (2)
C8—N1—C2—C3	179.0 (2)	C4—C5—C6—C1	−0.2 (3)
C8—N1—C2—C1	−0.1 (2)	N2—C5—C6—C1	−179.71 (19)
C6—C1—C2—C3	1.1 (3)	N4—N3—C7—C1	−179.81 (19)
C7—C1—C2—C3	−178.98 (19)	N4—N3—C7—C8	3.1 (3)
C6—C1—C2—N1	−179.75 (19)	C6—C1—C7—N3	2.1 (4)
C7—C1—C2—N1	0.1 (2)	C2—C1—C7—N3	−177.7 (2)
N1—C2—C3—C4	−179.6 (2)	C6—C1—C7—C8	179.7 (2)
C1—C2—C3—C4	−0.7 (3)	C2—C1—C7—C8	−0.1 (2)
C2—C3—C4—C5	−0.2 (3)	C2—N1—C8—O1	178.6 (2)
C3—C4—C5—C6	0.6 (3)	C2—N1—C8—C7	0.0 (2)
C3—C4—C5—N2	−179.8 (2)	N3—C7—C8—O1	−1.1 (4)
O2—N2—C5—C4	179.7 (2)	C1—C7—C8—O1	−178.6 (2)
O3—N2—C5—C4	−0.2 (3)	N3—C7—C8—N1	177.6 (2)
O2—N2—C5—C6	−0.7 (3)	C1—C7—C8—N1	0.1 (2)
O3—N2—C5—C6	179.4 (2)	N3—N4—C9—N5	1.3 (3)
C2—C1—C6—C5	−0.7 (3)	N3—N4—C9—S	−177.56 (15)

### Hydrogen-bond geometry (Å, °)

**Table:**

*D*—H···*A*	*D*—H	H···*A*	*D*···*A*	*D*—H···*A*
N4—H5···O1	0.93 (2)	2.08 (2)	2.791 (2)	132.6 (19)
N5—H6···O1^i^	0.83 (2)	2.13 (3)	2.957 (2)	173 (2)
N5—H7···O2^ii^	0.90 (3)	2.36 (3)	3.215 (3)	160 (2)
N1—H4···S^iii^	0.88 (3)	2.45 (3)	3.3123 (18)	170 (2)

Symmetry codes: (i) −*x*−1, *y*−1/2, −*z*+1/2; (ii) −*x*+1, −*y*, −*z*+1; (iii) −*x*−1, *y*+1/2, −*z*+1/2.
